# Customized middleware experience in a tertiary care hospital hematology laboratory

**DOI:** 10.1016/j.jpi.2022.100143

**Published:** 2022-09-24

**Authors:** Kristine Roland, Jim Yakimec, Todd Markin, Geoffrey Chan, Monika Hudoba

**Affiliations:** Vancouver General Hospital, Vancouver, BC, Canada

**Keywords:** Middleware, Autoverification, Hematology laboratory, Laboratory workflow

## Abstract

**Background:**

In the clinical laboratory, middleware is a software application that sits between the analyzer and the laboratory information system (LIS). One of the more common uses of middleware is to perform more efficient result autoverification than can be achieved by the LIS or analyzer alone. In addition to autoverification, middleware can support highly customized rules to handle samples and results from specific patient locations. The objective of this study was to review the impact of customized middleware rules that were designed and implemented in the hematology laboratory of a 1000-bed tertiary care adult academic center hospital.

**Methods:**

Three novel initiatives using middleware rules to achieve workflow efficiencies were retrospectively reviewed over different audit periods: preliminary neutrophil resulting for oncology patients, microcytosis interpretive comments, and 1 white blood cell differential (WBCD) reported per day. In addition, autoverification rates for complete blood count and differential (CBCD) and coagulation tests were calculated.

**Results:**

A preliminary neutrophil count was released from middleware on average 64 min before the final CBCD for Leukemia/Bone Marrow Transplant (L/BMT) outpatients, and on average 59 min earlier for oncology patients. Reflexing interpretive comments for select instances of microcytosis removed on average 500 slides per month from technologist review with an estimated cost savings of approximately $3383.33 CAD per month. The 1 WBCD per day rule resulted in a 5.1% cancelation rate, resulting in an estimated monthly cost savings of $943.46 CAD in reagents and technologist time. Finally, middleware rules achieved very high autoverification rates of 97.2% and 88.3% for CBC and CBCD results, respectively.

**Conclusions:**

Implementation of customized middleware hematology rules in our institution resulted in multiple positive impacts on workflow, achieving high autoverification rates, reduced slide reviews, cost savings, and improved standardization.

## Background

With increasing demands on productivity and decreasing resources, clinical laboratories are looking for ways to increase efficiency while maintaining accuracy and consistency of reported results. In high volume laboratories, middleware can be a useful tool for optimizing specimen handling and results reporting by virtue of highly customizable rules.

Middleware is a software application that sits between laboratory instrumentation and the laboratory information system (LIS). It can perform a variety of functions to assist technical staff such as autoverification of test results, holding and flagging results that may require additional action (e.g. failed delta check, critical value, results outside of range of the instrument), and quality control (QC) monitoring.^1^ Although an acceptable rate of autoverification can be achieved by having the autoverification algorithm fully defined in the LIS, the use of a middleware solution can further increase that rate. The sheer number of data elements (patient, specimen, test, with the ability to create end user defined elements for each type) that can be leveraged is significantly higher than what an LIS can offer. Also, there are additional locations within the middleware data stream where rules can be written than in an LIS alone.

In the clinical pathology literature, publications on middleware have largely focussed on improvements to laboratory test autoverification rates.^2^^,^^3^ However, the potential scope of middleware is much broader in that middleware-built rules can be designed to cancel redundant tests, append interpretive comments when pre-specified criteria are met, and reflex further testing (e.g. reruns, add-on testing, specimen routing). There is little published literature on how individual laboratories have leveraged these latter capabilities.

We implemented middleware in our Hematology laboratory in February 2011, and over the last decade we sought to design highly customized rules to not only improve our autoverification rates but also to improve workflow, turn around time (TAT), and our ability to manage increasing test volumes. Here we report a retrospective review of our autoverification rates as well as 3 of our novel customized middleware algorithms to determine their impacts on workload and cost savings.

## Materials and methods

### Setting

Our Hematology laboratory is located in a 1000-bed tertiary care academic adult hospital. Major inpatient services include general medical and surgical services as well as emergency, trauma and burns, critical care, cardiothoracic surgery, solid organ transplant, and leukemia/bone marrow transplant. In addition, our laboratory processes outpatient blood samples from the neighboring Cancer Centre.

Currently, the Hematology laboratory performs around 340 000 complete blood counts (CBC) and complete blood counts with differential (CBCD), and 5100 body fluids per year using Sysmex XN9000 hematology analyzer, with addition of automated digital white blood cell (WBC) differential and morphology analyzer CellaVision DI-60 (Sysmex America, Inc., Illinois, USA). Routine coagulation tests consisting of prothrombin time (PT), activated partial thromboplastin time (aPTT), fibrinogen, D-dimer, and thrombin time is 250 000 annually performed on ACL TOP 700 CTS by Instrumentation Laboratory (A Werfen Company, Bedford, MA). All instruments are interfaced to the LIS (Sunquest Laboratory version 6.4 and 10) through the middleware Data Innovations Instrument Manager (DI IM) (version 8.17, Colchester, Vermont, USA). CBCD parameters measured include 6-part WBC differential, nucleated red blood cell count (NRBC), reticulocyte count (RET), and immature reticulocyte fraction (IRF). Reticulocyte parameters are discrete and performed only if ordered.

### Middleware

**Fig. 1:**
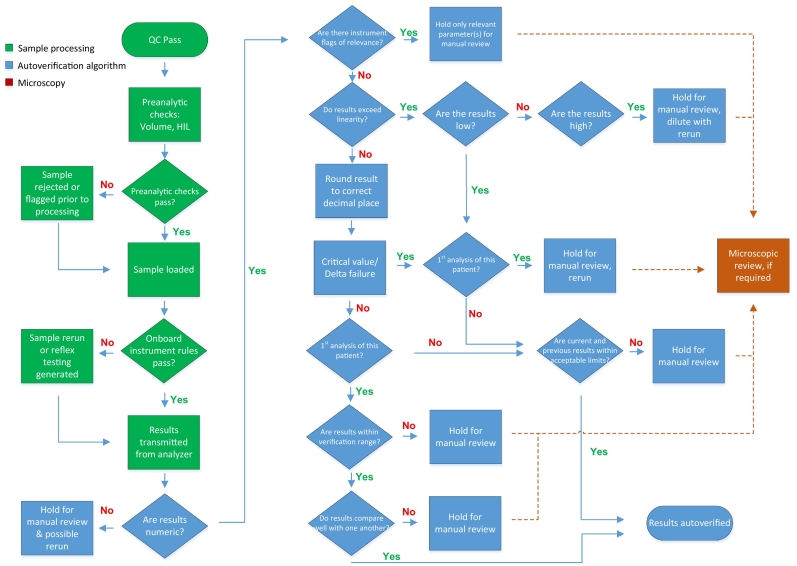
Middleware-driven hematology workflow.

The implementation of the middleware occurred on February 23, 2011. The autoverification rules algorithm along with rules for automated technologist comments and pathologist interpretive reports were created to ensure consistency and accuracy (Table 1). Peripheral blood, body fluid, and sputum keyboards were created in IM in order to have as many technical and pathologist functions on the same platform as possible. Rules were written within the keyboard configurations to provide technologist guidance, calculate absolute differential counts, alert them to the presence of critical values, pathologist review criteria, and reflex a pathologist review order. In effect, the middleware rules dictate all specimen and results handling between pre-analytical specimen processing and microscopic slide review (Fig. 1).

**Table 1:** Middleware rules for complete blood count, differential and coagulation testing.

Rule source		Rule	Hold for review	Notes
*CBC and differential*				
DI		Sample collection time >24 h	CBC, Diff	Suppress Auto diff + RBC indices
DI		Sample collection time >72 h	Reticulocyte	Not reported
DI		Patient age <3 days		Reflex CBC, Diff, NRBC, Retic, Smear
DI	★	WBC <0.5	Diff	Reflex smear review + referral
DI		WBC <0.5 + previous WBC >1.0 + not oncology		Reflex smear review + referral
DI		WBC >30.0 + Outpatient		Reflex Diff
DI		WBC 250.0 – 450.0	Diff	Report RBC indices as Unavailable
DI		WBC exceeds linearity	WBC, HCT, Diff, Reticulocyte	Report RBC indices as Unavailable
DI		WBC lower limit of quantitation		Report WBC as < x.x
DI	★	Neutrophil # <1.0 + not oncology		Reflex smear review
DI	★	Neutrophil # <0.5		Follow Critical Result SOP + referral
DI	★	Neutrophil # >30.0		Reflex smear review
DI		Neutrophil # >50.0		Referral if no previous >50.0
DI		Lymphocyte # > reference interval Child		Reflex smear review + referral
DI	★	Lymphocyte # >5.5 Adult		Reflex smear review + referral
DI	★	Monocyte # >2.0 + Neutrophil # <8.0		Reflex smear review
DI	★	Monocyte # >3.0		Reflex smear review + referral
DI		Eosinophil % >20.0	Diff	Reflex smear review
DI	★	Eosinophil # >2.0		Reflex smear review + referral
DI	★	Basophil # >0.5	Diff	Reflex smear review + referral
DI	★	IG % >5, or >10 + previous <5, or >20 + previous <10		Reflex smear review Suppress IG # <0.2
DI	★	NRBC % >2.0 + not ICU/oncology		Reflex smear review + referral
DI		NRBC % >25.0 + patient age <31 d		Reflex smear review + referral
DI		NRBC linearity	WBC, Diff, NRBC	
Sysmex/DI		WBC abnormal scattergram + WBC >0.5	Diff	Reflex smear review
Sysmex/DI		Abnormal lymphocytes/blasts flag	Diff	Reflex smear review Oncology: Reflex Preliminary ANC
Sysmex/DI		Left shift flag + no previous results or new ED visit		Reflex smear review
Sysmex/DI	★	Atypical lymphocytes flag or new ED visit		Reflex smear review
DI		Differential vote-out		Suppress Auto diff, perform manual
DI		RBC linearity	RBC indices	Dilute X7
Sysmex/DI		RBC abnormal distribution + MCHC >375	All results	Reflex rerun and smear review
Sysmex/DI	★	Dimorphic population		Reflex smear review
Sysmex/DI		RBC agglutination	All results	Reflex rerun
DI		HB outside reference interval – Child		Reflex smear review. Refer <80
DI	★	HB <100 + not IDA + Outpatient / ED new admission		Reflex smear review
DI	★	HB <75 + not IDA + Inpatient		Reflex smear review
DI	★	HB <50 + not post-op / trauma / acute bleed / known	All results	Reflex smear review
DI	★	HB >160 female or >180 male		Reflex smear review
DI	★	HB critical		Reflex rerun. HB <50 or >230
DI		HB linearity	HB, MCH, MCHC	Dilute X7
DI		HB delta failure	All results	14 days: + 40 Adult, + 20 Child
Sysmex/DI		Turbidity/Hb interference + MCHC >375	CBC, Diff	Reflex rerun, Dilute X7
DI		HCT >0.55, add Patient User Field		For use in coagulation rules
DI		HCT linearity	CBC	Dilute X7
DI	★	MCV outside reference interval – Child		Reflex smear review + referral
DI		MCV delta failure	All results	60 days: + 5 Adult, + 4 Child
Sysmex/DI		MCV <60	PLT	Reflex PLT-F
DI		MCV <80 + RBC, HB, RDW, Age, Gender		Auto comments - Microcytosis
DI	★	MCV <80 + HB <50 or HB >165 male or >150 female		Reflex smear review and referral
DI	★	MCV 105-110 + HB <100 or PLT <50 or Neutrophil# <1.0		Reflex smear review and referral
DI	★	MCV >110		Reflex smear review Referral with exceptions
Sysmex/DI		MCHC <275 or > 375	All results	Reflex rerun
DI	★	PLT <100		Reflex smear review Referral if Child
DI		PLT <75 + previous >120	All results	Reflex smear review
DI	★	PLT <50	All results	Child – critical result Referral with exceptions
Sysmex/DI	★	PLT <20	All results	Reflex PLT-F Adult – critical result
DI		PLT >800 Child		Reflex smear review and referral
DI	★	PLT >1000 Adult		Reflex smear review and referral
DI		PLT linearity	PLT	Dilute X7 and reflex smear review
DI		PLT delta failure	All results	14 d: % delta is count-dependent
DI		PLT lower limit of quantitation		Report PLT as < x
DI		Citrate PLT	Citrate PLT	Add 10% and reflex smear review
Sysmex/DI		PLT abnormal scattergram		Reflex smear review
Sysmex/DI		PLT abnormal distribution + PLT <50		Reflex PLT-F
Sysmex/DI		PLT clumps + PLT <125 or >350	PLT	Reflex smear review
Sysmex/DI		PLT clumps + PLT <75	All results	Reflex smear review
DI		Reticulocyte linearity	Reticulocyte	Perform manual reticulocyte
Sysmex		Reticulocyte abnormal scattergram	Reticulocyte	Dilute X5
Sysmex/DI		Fragments		Reflex PLT-F and smear review
DI		If previous smear - Blast/Hairy cells/Megakaryocytes	Diff	Reflex smear review
DI		If previous smear - PLT clumping	PLT	Reflex smear review
DI		Specific patient – RBC Agglutination / Diff + NRBC / PLT	Specific test(s)	Reflex smear review
DI		One differential per day		Suppress subsequent Diff order(s)
DI		ICU – one smear review per day		
DI/LIS		Lab-use only test to trigger LIS-reflexed tests		eg: Pathologist review, Preliminary ANC, Smear review

*Coagulation*				
IL TOP/DI		Pre-analytic: Hemolyzed / Icteric / Lipemic samples	INR, PTT, D-Dimer, FIB	Auto append comment
IL TOP/DI		Pre-analytic: Lipemic and D-Dimer above cut off	D-Dimer	For ultra centrifugation
DI		Pre-analytic: HCT >0.55 + results above normal range	All results	For special collection
DI		Pre-analytic: manually prepared dilutions	Factor VIII, IX	Apply dilution factor
IL TOP/DI		Analytic: specific instrument warnings + errors	That test	Add technologist guidance
DI		Sample collection time >4 h + PTT above normal range	PTT	Confirm collection date/time
DI		Sample collection time >12 h + PTT within normal range	PTT	Confirm collection date/time
DI		Sample collection time >24 h	INR	Reported as too old
DI		Sample collection time >72 h	FIB, D-Dimer, TT	Reported as too old
IL TOP/DI		Clotting test < test range (INR, PTT, TT, FIB)	All results	Auto repeated
IL TOP/DI		Clotting test > test range (INR, PTT, TT, FIB)	That test	Auto repeated
DI		Lower + Upper reportable limits		Reported as < xx.x or > xx.x
DI		Delta failure		Auto repeated
DI		INR 3.1-6.0 + Hemodialysis location		Reflex TT + heparin-neutralized INR
DI		INR 3.6-6.0 + Outpatient	INR	Reflex Phone call
DI		INR >4.5 + no previous within 36 h	INR	
DI		INR >6.0 Critical	INR	Auto repeated + Phone call
DI		INR delta failure	INR, PT	36 h: Absolute value delta
DI		Research INR & PT		Append MNPT and ISI
DI		PTT >48 + no previous within 7 days	PTT	
DI		PTT >110 + previous result normal within 7 days	PTT	
DI		PTT > defined phone value	PTT	
DI		PTT >48 isolated + INR / TT normal		Reflex Lupus-insensitive PTT + Referral
DI		PTT delta failure	PTT	24 h: Absolute value delta
DI		D-Dimer		Append interpretational comment
DI		FIB <1.0 + previous >1.0 or no previous result	All results	
DI		FIB <0.6 Critical	All results	Reflex referral
DI		FIB delta failure	FIB	48 h: + 50%
DI		Add Pathologist Referral result field		

★ **denotes if no previous test result** CBC: Complete blood cell count, WBC: White blood cell count, IG: Immature granulocytes, RBC: Red blood cell count, HB: Hemoglobin [g/L], HCT: Hematocrit, MCV: Mean cell volume [fL], MCHC: Mean cell hemoglobin concentration [g/L], RDW: Red cell distribution width, NRBC: Nucleated red blood cell count, PLT: Platelet, ANC: Absolute neutrophil count, ED: Emergency department, IDA: iron deficiency anemia, INR: International normalized ratio, PT: Prothrombin time (s), PTT: Activated partial thromboplastin time (s), FIB: Fibrinogen, quantitative (g/L), TT: Thrombin time (s), MNPT: Mean normal prothrombin time, ISI: International sensitivity index, Referral: Pathologist review, Smear review: Technologist review.

**Fig. 2:**
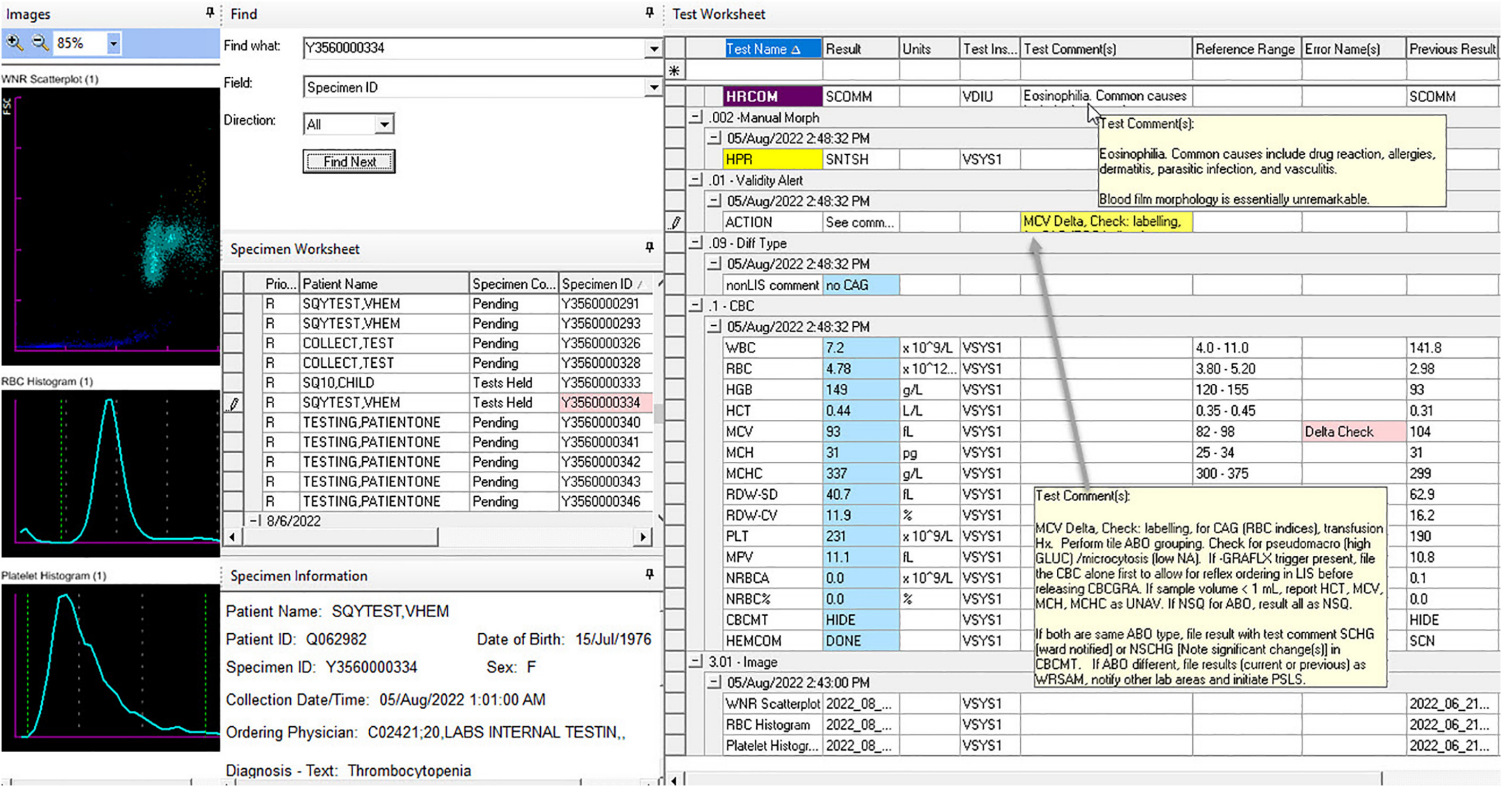
IM middleware hematolgy workspace.

The workspaces within the middleware are fully customizable. With the ability to use both pre-defined and free text coded entries, we were able to configure a hematology workspace application for reporting blood film, fluid morphology, and coagulation interpretations within IM. This module provides information on recent consecutive CBCs, instrument flags, technologist reason for referral, Sysmex scatterplots, and clinical diagnostic information provided in LIS (Fig. 2). No LIS enhancements were required, however we did request analyzer driver enhancements to capture specific data elements, as is commonly required from many middleware customers. We also requested the ability to edit comments (both pre-defined and free text) which allowed for pathologist workflow to be incorporated onto the platform. Onboarding all these functions into middleware reduced reliance on paper printouts and created an essentially paperless system. The writing and maintenance of all middleware rules remains under the autonomy of the Hematology laboratory.

### Customized middleware algorithms

The following algorithms were built using customized middleware rules and were selected for this retrospective analysis:

#### Preliminary neutrophil reporting

Our outpatient leukemia and bone marrow transplant (L/BMT) and Oncology physicians requested a preliminary neutrophil result before the full CBCD is resulted (in the event of a flagged differential that fails autoverification), in order to initiate chemotherapy treatment as quickly as possible. This was achieved by first building a new LIS trigger code to reflex order a preliminary neutrophil count. Then a rule was written within the middleware to limit the test by patient location (L/BMT clinic and Oncology clinic) and by the presence of WBC differential flags (such as the blast/abnormal lymph flag and abnormal scattergram flag). The preliminary neutrophil result is displayed as such and the final neutrophil value is resulted with the CBCD.

#### Microcytosis interpretive comments

As a sole abnormality, the differential diagnosis of microcytosis with or without anemia is limited. We created interpretive comments in the middleware specific to the mean cell volume (MCV), hemoglobin, red blood cell count, red cell distribution width-coefficient of variation (RDW-CV), se,x and age of the patient. Based on these parameters, 1 of 6 interpretive comments is automatically appended to the CBC result by the middleware and a slide is not generated (unless there is another concurrent flag requiring slide review). The intent was to reduce slide reviews by both technologists and pathologists on a common but low-stakes finding on a CBC.

#### One WBC differential per day

After consultation with stakeholder physicians at our institution, it was agreed that a WBC differential did not need to be repeated on a patient within 1 calendar day, even if a repeat CBCD was ordered. The one exception was the context of autologous stem cell transplant collections, where a pre-/post-collection WBC differential was required for quality assurance purposes. We created a rule within the middleware to cancel a repeat same-day WBC differential, except for samples from autologous stem cell collections. This rule was written at the point of order download from the LIS to the middleware, so that the differential would not be run. Instead a comment would be appended to the CBC stating: “*One differential reported per calendar day. See previous differential*”. Full details of this project are explained elsewhere.^4^

#### Autoverification rates

We created autoverification rules in the middleware (as well as LIS when appropriate) for CBC, CBCD, and coagulation tests. Our routine coagulation testing includes five parameters: aPTT, PT, thrombin time (TT), fibrinogen, and D-dimer. Autoverification is achieved when the middleware releases results into the LIS without holding them due to a programmed rule.

For this review of the above algorithms, 3 audit periods were selected based on respective test volumes. A short time period (September 2, 2021–September 15, 2021) was selected to collect autoverification rates on high volume tests (i.e. CBC and coagulation tests). An intermediate audit period (September 1, 2021–December 31, 2021) was selected to collect preliminary neutrophil reporting times, and the period of January 1, 2021–December 31, 2021 was selected to collect microcytosis interpretive comments and WBC differential cancelations. Data was extracted from the DI Instrument Manager and Sunset Laboratory databases (Oracle Corp. Austin, Texas). A Microsoft Excel spreadsheet was used for statistical analysis.

## Results

### Preliminary neutrophil reporting

During the 4-month audit period, there were a total of 948 CBCD tests reported with a preliminary neutrophil result (Table 2). Most of these CBCD tests were from L/BMT outpatients (806) while a smaller proportion were from the neighboring cancer clinic (142). The TAT for laboratory results is longer for cancer patients than for L/BMT outpatients due to sample transport time; the oncology clinic is 2 blocks away from the main building housing both the Hematology laboratory and L/BMT clinic. Although there is a significant range in reporting times due to the presence of different CBCD flags, on average a preliminary neutrophil result is released 64 min before the full CBCD for L/BMT outpatients and 59 min earlier for oncology patients.

**Table 2:** Time to release complete blood cell counts and preliminary neutrophil counts during audit period.

	Time to CBCD result release		Time to preliminary neutrophil result release		Average time saved (min)
	Average (min)	Range (min)	Average (min)	Range (min)	
L/BMT outpatients (n = 806)	90	34 – 240	26	6 – 87	64
Cancer patients (n = 142)	127	70 – 273	68	34 – 87	59

CBCD = complete blood count with white blood cell differential; L/BMT = leukemia and bone marrow transplant; min = minutes.

### Microcytosis interpretive comments

During the 1-year audit period, there were 6263 microcytosis interpretive comments automatically appended to CBC results by the middleware. Table 3 shows the distribution of interpretive comments and the criteria for each. Of these, 265 (4.2%) still met slide review criteria due to other flags, initiating a slide review by the technologist, and of these 154 (2.5%) met criteria for Pathologist review. However, in the remaining 5998 cases, slides were not generated for manual review, which equates to a reduction of approximately 500 slides per month. This results in an estimated 5000 min (83.3 h) of technologist time saved monthly (based on slide preparation and manual review of approximately 10 min of technologist time per slide). At a rate of $0.47 CAD for slide materials and $37.78 CAD technologist time per hour, there is a monthly estimated cost savings of approximately $3383.33 CAD per month.

**Table 3:** Interpretive comments automatically appended in middleware based on complete blood count parameters.

Sex	Hb	RBC	MCV	RDW	Comment	Total (n = 6263)
F	<120	<4.50	<55	>15.8	Microcytic anemia suggestive of iron deficiency.	4
M	<130	<4.80				
F	<120	<4.50	55–70	>15.8	Microcytic anemia. Common causes include iron deficiency or thalassemia.	313
M	<130	<4.80				
F	<120	<4.50	70–80	>15.8	Microcytic anemia. Common causes include iron deficiency, anemia of chronic disease, or less likely thalassemia.	1699
M	<130	<4.80				
F	Any	>4.90	<70	<15.8	Microcytic red blood cell morphology. Common causes include thalassemia trait, or less likely iron deficiency.	268
M		>5.20				
F	Any	>4.90	70–80	<15.8	Microcytic red blood cell morphology. Common causes include thalassemia trait, or less likely iron deficiency or anemia of chronic disease.	844
M		>5.20				

For cases where above criteria are not met, the following comments are used:						
Any	Any	Any	<55		Red blood cell microcytosis, likely due to iron deficiency.	1
			55–70		Red blood cell microcytosis, consider iron deficiency or thalassemia.	834
			70–80		Red blood cell microcytosis, consider iron deficiency, anemia of chronic disease, or thalassemia trait.	2300

F = female; M = male; Hb = hemoglobin; RBC = red blood cell count; MCV = mean corpuscular volume; RDW = red cell distribution width.

### One WBC differential per day

With an average of 18 786 CBCD ordered per month, the number of canceled WBC differentials was on average 952 (range 893–1007; ±35.3SD) (Table 4). This equates to a cancelation rate of 5.1% (range 4.8–5.6%; ±0.3SD) during the 1-year audit period. At an estimated cost of $0.33 CAD per differential in reagents, this resulted in a cost savings of approximately $314.16 CAD per month (based on average 952 canceled differentials per month). In addition, some of these canceled differentials would have generated a slide review. Given our historic rate of 9.8% for flagged WBC differentials, the estimated technologist review avoidance was 93 slides per month. This equates to 930 min (or 15.5 h) of technologist time saved monthly, and a monthly savings of $629.30 CAD (using same cost analysis as for microcytosis interpretive comments).

**Table 4:** Monthly canceled white blood cell differentials due to one differential per day rule.

Month in 2021	Total CBCD ordered	WBC differentials canceled	%
January	19 121	937	4.9
February	18 284	907	5.0
March	20 401	917	4.5
April	19 501	963	4.9
May	20 762	1004	4.8
June	19 210	965	5.0
July	18 255	979	5.4
August	18 385	893	4.9
September	17 978	951	5.3
October	18 065	1007	5.6
November	18 023	951	5.3
December	17 450	946	5.4

Average	18 786	952	5.1

### Autoverification rates in CBCD and coagulation

The overall rate of CBC autoverification was 97.2% (Table 5). Of the CBC that failed autoverification, the vast majority had all results held; only 0.1% had only Platelet result held due to a platelet clumping suspect flag on platelet results outside of the normal range. The reasons for holding all results were varied, the most frequent being mean corpuscular volume (MCV) delta check (1.0%).

**Table 5:** Autoverification rates for complete blood counts and coagulation tests during audit period.

Parameter	Total	Autoverification rate (%)
Total CBC and CBCD performed	13 414	
Number of CBC and CBCD with all results autoverified	13 036	97.2

Total WBC differentials performed	9263	
Number of differentials canceled due to low WBC	222	2.4
Number of differentials canceled due to one diff/day	435	4.7
Number of remaining differentials autoverified	7597	88.3

Number of reticulocytes performed	291	
Number of reticulocytes autoverified	265	91.1

Number of INR performed	4447	
Number of INR autoverified	4349	97.8

Number of PTT performed	3874	
Number of PTT autoverified	3658	94.4

Number of quantitative fibrinogen performed	513	
Number of quantitative fibrinogen autoverified	488	95.1

Number of TT performed	91	
Number of TT autoverified	78	85.7

Number of D-dimer performed	325	
Number of D-dimer autoverified	320	98.5

CBC = complete blood count; CBCD = complete blood count with differential; WBC = white blood cells; diff = differential; INR = international normalized ratio; PTT = partial thromboplastin time; TT = thrombin time.

Of all the CBCD, 7.1% of the WBC differentials were canceled due to existing rules (i.e. low WBC count or 1 differential per day). Of the uncanceled CBCD, the differential autoverification rate was 88.3%. The reasons for holding the differential result were varied, but the most common was the blast/abnormal lymph flag (5.3%). The rate of technologist slide review/manual differential was 8.9% and the rate of Hematopathologist slide referral was 1.5%.

The autoverification rate for reticulocyte count was 91.1%. The most common reason for holding the reticulocyte result was an abnormal scattergram flag (8.3%).

The autoverification rates for aPTT, PT, TT, fibrinogen, and D-dimer were 94.4%, 97.8%, 85.7%, 95.1% and 98.5%, respectively. In all cases, the TT time was held because of failed clot curve (i.e. no clot within acquisition time). The PT, aPTT and fibrinogen results were held for a variety of reasons. The most common reason for holding D-dimer was QC failure (0.9%).

## Discussion

Our retrospective analysis of customized middleware algorithms in a Hematology laboratory demonstrates how middleware capabilities can be expanded over and above autoverification of laboratory test results. Comprehensive rules written in middleware can streamline and standardize Hematology laboratory operations including redundant test cancelation, preliminary result reporting, and interpretive comments that is specific to different hospital locations.

Most of the published literature to date is limited to autoverification rules written in the hematology analyzer and the LIS.^5^, ^6^, ^7^, ^8^, ^9^, ^10^, ^11^, ^12^ Reported autoverification rates for CBC results have ranged from 63% when rules were built in the analyzer^10^ to 81% when written in LIS.^12^ Similarly in coagulation, reported autoverification rates have ranged from 65% to 82%.^5^^,^^10^ High rates of LIS-based autoverification were achieved in an outpatient hematology/coagulation laboratory; however, outpatient samples may be less complex to result than predominantly inpatient population.^6^^,^^7^ We were able to find 1 report of a hematology laboratory that built autoverification rules in middleware and these authors used similar instrumentation and middleware as our laboratory.^13^ They achieved an autoverification rate of 93.5% for CBC and 89.9% for individual CBC components, which was similar to our results of 97.2% for all CBCD and 88.3% for WBC differentials.

Our review of novel middleware-built algorithms demonstrate that the capabilities of middleware extend far beyond autoverification. Two of our initiatives (1 WBC differential per day rule and standardized microcytosis comments) were successful in reducing manual slide review which saved technologist (and sometimes pathologist) time. Other authors have aimed to reduce unnecessary or redundant laboratory tests by focusing on clinician ordering practices using educational methods however results tend to be modest and temporary.^14^, ^15^, ^16^, ^17^, ^18^ Our approach using middleware has been sustainable with no reduction in effect over time. Finally, we showed that a preliminary neutrophil count can be released on average 1 h before a flagged CBCD is fully resulted, which can improve clinical management of hematology/oncology patients without additional workload on technologists.

Finally, there is a significant benefit to having the hematology rules engine under the autonomy of the Hematology laboratory. This self-sufficiency allows the technical leadership to modify the algorithms in real time, rather than submitting change requests to a heavily burdened LIS department and waiting in queue. In fact within our region, this 1 middleware solution has since expanded for use at multiple sites in multiple disciplines (Chemistry, Autoimmune testing, Microbiology). The LIS department supporting these multiple sites has now embraced it to interface all new analyzers.

There are limitations to using middleware. There is the cost of initial capital output for the purchase of the production and test servers, connections, interfaces, and rules writing course. The initial build and validation of the rules is time-consuming, and requires a certain level of expertise among technical staff. Regular validation of rules is recommended in accordance with regulatory and accreditation requirements.

## Conclusion

Middleware offers a flexible platform for laboratories to achieve standardized, efficient results reporting in a paperless environment. High autoverification rates using highly customized rules can be achieved for complex laboratory tests with multiple analytes such as the CBCD. In addition, laboratories can create their own context-specific rules to achieve targeted goals including, but not necessarily limited to, canceling redundant tests, appending interpretive comments, and releasing preliminary results. Using middleware to its full potential can improve workflow and result in cost savings. The use of middleware to create customized rules appears to be under-represented in the literature, and may indicate that this technology is not being used to its full potential.

## Funding

This research did not receive any specific grant from funding agencies in the public, commercial, or not-for-profit sectors.

## Declaration of Competing Interest

The authors declare that they have no competing interests.
